# The effects of electronic cigarette use patterns on health-related symptom burden and quality of life: analysis of US prospective longitudinal cohort study data

**DOI:** 10.3389/fpubh.2024.1433678

**Published:** 2024-11-13

**Authors:** Yue Cao, Xuxi Zhang, Ian M. Fearon, Jiaxuan Li, Xi Chen, Yuming Xiong, Fangzhen Zheng, Jianqiang Zhang, Xinying Sun, Xiaona Liu

**Affiliations:** ^1^Department of Health Sciences, Smoore Tech Research Institute, Shenzhen, China; ^2^Department of Social Medicine and Health Education, School of Public Health, Peking University, Beijing, China; ^3^whatIF? Consulting Ltd., Harwell, United Kingdom

**Keywords:** electronic cigarette, tobacco, cigarette smoking, harm reduction, symptom burden, quality of life

## Abstract

**Objective:**

This study aimed to examine the association between e-cigarette (EC) use patterns and health-related symptoms (fatigue, pain, and emotional problems) as well as general quality of life (QoL).

**Methods:**

Data were analyzed from 7,225 adults across Waves 1–6 of the US Population Assessment of Tobacco and Health (PATH) study. Current combustible cigarette (CC) or EC use patterns included dual CC/EC use, exclusive EC use, non-current use of CC or EC, and exclusive CC smoking. Multivariate linear mixed-effects models were used to investigate longitudinal associations between EC use patterns, and symptom burdens/QoL scores.

**Results:**

Those who were not currently smoking or vaping reported the lowest fatigue, pain, and emotional problems, and the best QoL, among the four groups (all *p* < 0.001). Compared to exclusive CC smoking, exclusive EC use was associated with a significant decrease of 0.065 units in average fatigue (95% confidence interval [CI]: −0.121, −0.009), of 0.206 units in average pain (95% CI: −0.355, −0.058), and of 0.103 units in average QoL scores (95% CI: −0.155, −0.051), with emotional problems similar over time.

**Conclusion:**

Exclusive EC users had less health-related symptoms and better QoL than those who were exclusive CC smokers. This should be taken into account when assessing the harm reduction potential of ECs.

## 1 Introduction

Despite decades of tobacco control efforts to reduce smoking-related harms, combustible cigarette (CC) smoking is still the leading global cause of death and disease. Without further efforts to reduce the prevalence of cigarette smoking, it is predicted that smoking may cause up to a billion deaths this century (1). Cigarette smoking is a recognized risk factor for cardiovascular disease, chronic obstructive pulmonary disease (COPD) and lung cancer, along with many other human diseases (2–4). Nicotine, a chemical released from tobacco during smoking, is not considered to be responsible for smoking-related disease, although its use is known to be addictive (5). Instead, smoking-related diseases are primarily initiated by prolonged inhalational exposure to the numerous chemical toxicants found in cigarette smoke (5–8), which are formed during the process of combustion and have established links to human diseases (9).

Electronic cigarettes (e-cigarettes; ECs) are battery powered devices which heat a liquid solution, commonly containing nicotine, to produce an inhalable vapor (10). Since EC liquids do not contain tobacco, and also since the process of vapor formation involves heating and not combustion, EC vapor contains far fewer chemical toxicants than cigarette smoke, and those that are present are found at significantly lower levels (11–13). In smokers who completely switch to using ECs, both cross-sectional and longitudinal studies have demonstrated that toxicant exposure is significantly reduced, and toxicant exposure among those who have switched can approach levels seen either with smoking cessation or among non-smokers (14–20). Thus, ECs are a reduced exposure alternative for cigarette smokers who completely switch to using them and may help to reduce smoking-related disease risk (20–28). Increasing evidence suggests that ECs can assist with smoking cessation (29–34), and this is supportive of a potential role for ECs in tobacco harm reduction efforts. However, some evidence supports that smokers do not necessarily completely switch immediately, and the transition to EC use may involve a transition period of dual use (35). Such dual use, while potentially being a stepping stone between smoking reductions and complete smoking cessation depending on the particular CC/EC use patterns of the individual (36–39)may still give rise to some degree of toxicant exposure (18, 19, 40, 41) and therefore health risk (42).

While smoking is associated with a significantly increased risk of developing smoking-related disease, smoking cessation can, in the short-term, lead to reductions in quality of life (QoL) and this may promote relapse and lead to failed cessation attempts (43). However, those smokers who, during a cessation attempt, experience the largest increases in QoL factors such as physical functioning, general health and vitality may be more likely to remain abstinent long-term (43). In addition, scores for these QoL domains, as well as other domains including mental health and bodily pain, are higher among former smokers than among current smokers (44). Furthermore, some QoL domain scores, particularly those related to symptoms and the impact of smoking on QoL, were improved among former smokers compared with current smokers (45). Therefore, as well as reducing disease risk, quitting smoking also enhances QoL (46) and this should be taken into account when attempting to determine the harm reduction potential of products such as ECs which may assist with smoking cessation.

Few studies have examined changes in QoL in smokers who switch to using reduced risk tobacco and nicotine products such as ECs, and there are also limited data regarding the long-term impact of different CC/EC use patterns on QoL among adult individuals. Using data from the United States (US) Population Assessment of Tobacco and Health (PATH) survey study, Price et al. (47) assessed differences in QoL among adults with a previous self-reported cancer diagnosis who were either current smokers or current EC users. This cross-sectional analysis found that current smokers experienced greater fatigue, pain, emotional problems and general QoL than former or never smokers, and also that current EC use was associated with greater fatigue, pain, and emotional problems, but not general QoL, compared with non-use (47). However, that study did not examine differences in QoL and associated factors between smokers who had switched to using ECs or remained smoking, nor did they assess changes in QoL over time. Another cross-sectional analysis, using data from a survey study which administered an abbreviated version of the World Health Organization Quality of Life instrument (WHOQOL-BREF) to college students who used ECs or smoked cigarettes, demonstrated that exclusive cigarette smokers had significantly lower general QoL scores, as well as reduced scores for psychological, social relation and environmental health factors, than tobacco and nicotine product non-users (48). In addition, this study also found that scores in these domains, as well as physical health, were not significantly different between EC users and tobacco/nicotine non-users (48). Again however, the cross-sectional design used in that study did not assess either changes in QoL over time among cigarette smokers switching to EC use or differences between EC users and smokers. In contrast, a recent longitudinal study using the same questionnaire did not observe changes in QoL among smokers switching to EC use, despite robust reductions in cigarette consumption (49). This may be explained by the short time frame of the study, and it is of note that QoL also did not change among smokers undergoing a more traditional smoking cessation program (49). Another longitudinal assessment of physical function, a factor of importance to QoL, using a 6-min walk distance (6MWD) test among smokers with COPD who switched to using either ECs or heated tobacco products (50–52) did however observe significant improvements in 6MWD.

Since CC smoking reduces QoL, long-term smoking abstinence improves QoL, and QoL is not different between e-cigarette users and non-users, the aim of this study was to assess differences in QoL among individuals with different cigarette/EC use patterns. Using data from the US nationally-representative PATH survey study, we examined QoL factor scores in adults who were current exclusive EC users, dual CC/EC users, or non-current users of CC or EC, and compared these with QoL factor scores among those who were current exclusive CC smokers. The findings of this analysis may be of importance when considering the harm reduction potential of EC use.

## 2 Methods

### 2.1 Data source and study sample

The PATH study is a nationally representative longitudinal cohort study of adult and youth tobacco use patterns and behaviors and health outcomes in the US (53). The PATH study used a four-stage stratified area probability sample design in Wave 1, consisting of a stratified sample of geographical primary sampling units (PSUs) at the first stage, smaller geographical segments at the second stage, residential addresses located in the segments at the third stage, and households within the residential addresses at the last stage. As part of the complex sample design, survey weights for participants were constructed to compensate for variable probabilities of selection, differential non-response rates, and possible deficiencies in the sampling frame. The first wave of the PATH study was conducted between 2013 and 2014, Wave 2 was conducted between 2014 and 2015, Wave 3 was conducted between 2015 And 2016, Wave 4 was conducted between 2016 and 2018 with a replenishment sample to supplement the Wave 1 sample, Wave 5 was conducted between 2018 and 2019, and Wave 6 was conducted during 2021 (between March 2021 and November 2021). Our analyses included 7,225 adult respondents who completed all the interviews from Wave 1 to Wave 6.

### 2.2 Study variables

#### 2.2.1 Tobacco exposures of interest

Adult respondents reported lifetime and current use of combustible cigarettes (CCs) and ECs at each wave. Participants were considered as current established CC smokers at a specific wave if they had smoked at least 100 cigarettes in their lifetime and currently smoked CCs every day or some days. Similarly, they were characterized as current established EC users if they had used ECs fairly regularly in their lifetime and currently used ECs every day or some days. Respondents were divided into four mutually exclusive categories according to their current CC and EC use status at a given wave as follows: (1) exclusive CC smokers (current established CC smokers but not current established EC users); (2) dual users (current established CC smokers and current established EC users); (3) exclusive EC users (current established EC users but not current established CC smokers); (4) non-current CC or EC users (neither current established CC smokers or current established EC users).

#### 2.2.2 Sociodemographic variables

The following covariates were selected for this analysis: age (18–24, 25–44, or 45 years and older), gender (male or female), race/ethnicity (Hispanic, non-Hispanic White, non-Hispanic Black, or others), education level (less than Bachelor’s degree or Bachelor’s/advanced degree), employment status (full-time, part-time, or unemployed), total household income in the past 12 months (less than $50,000 or $50,000 or more), health insurance coverage (yes or no), and visit to an emergency room or urgent care center for a health problem of respondents themselves in the past 12 months (yes or no). Missing data on age, gender, race/ethnicity and education were imputed following the PATH Study User Guide (54). All covariates used in longitudinal analyses are from the earlier wave of each wave pair.

### 2.3 Symptom burden

PATH survey questions relevant to symptom burden contained single-item assessments of fatigue, pain, and emotional problems adapted from validated questionnaires in the PROMIS suite of measures, as follows.

#### 2.3.1 Fatigue

Respondents were asked to rate their fatigue (“feeling unrested or overly tired during the day, no matter how many hours of sleep you have had”) on average in the past 7 days on a scale of 1 to 5, with 1 being no fatigue and 5 being very severe fatigue.

#### 2.3.2 Pain

Respondents were asked to rate their pain on average in the past 7 days on a scale from 1 (“no pain”) to 10 (“the worst pain imaginable”).

#### 2.3.3 Emotional problems

Respondents were asked how often they have been “bothered by emotional problems such as feeling anxious, depressed, or irritable” in the past 7 days on a scale of 1 (“never”) to 5 (“very often”).

#### 2.3.4 Quality of life

Respondents were asked to rate their quality of life (QoL; “feeling unrested or overly tired during the day, no matter how many hours of sleep you have had”) in general on a scale from 1 (excellent) and 5 (poor), with higher scores indicating worse QoL.

### 2.4 Statistical analyses

Unweighted values as well as percentages and weighted percentages were reported for socio-demographic characteristics for the sample at Wave 1 of the PATH Study. The 100 balanced repeated replicate (BRR) weights with Fay’s correction factor of 0.3 were used to calculate the 95% confidence intervals (CIs) for the weighted percentages. Weighted prevalence was estimated for each type of current use behavior (exclusive CC smoking, dual use of CC and EC, exclusive EC use, and non-current use of both) for respondents at each baseline wave. All-wave sampling weights were included when estimating the prevalence to account for the complex survey design and to represent the whole U.S. population.

Four linear mixed-effects regression (LMER) models were established to investigate the longitudinal association between CC or EC use assessed at each baseline wave and symptom burdens as well as QoL scores at each follow-up wave over five 2-wave periods (Wave 1 to Wave 2, Wave 2 to Wave 3, Wave 3 to Wave 4, Wave 4 to Wave 5, and Wave 5 to Wave 6), with a random intercept for each individuals and each 2-wave period. The LMER analyses were weighted using the Wave 6 longitudinal (all-waves) full-sample and replicate weights for the Wave 1 cohort. Least-squared mean differences in fatigue, pain, emotional problems and QoL between the exclusive CC smoking group and other groups (dual use, and exclusive EC use, non-current use) were reported with 95% CIs. Participants who were missing symptom burdens or QoL data, CC or EC use data, or covariate data were omitted from analyses (<1%). *p* values <0.05 were considered statistically significant. All statistical analyses were conducted using R Statistical Software (version 4.3.0 for Windows).

## 3 Results

### 3.1 Demographics

Brief sociodemographic characteristics of the 7,225 participants whose data were analyzed in this study are presented in Table 1. Approximately half of the selected respondents were male, and the majority were aged 25 years or older. The analytic sample were predominantly of White race and not Hispanic. The majority were educated to a level lower than Bachelor’s degree level, worked full-time at least 35 h per week, and had a total household income of less than $50,000. Three-quarters of respondents had health insurance coverage, and a third had visited an emergency room or urgent care center in the past 12 months for a health problem of their own.

**Table 1:** Sociodemographic characteristics.

	Overall *N* = 7,225	
	*N* (%)	Weighted % (95% CI)
Gender		
Male	3,470 (48.0)	53.3 (51.9, 55.0)
Female	3,755 (52.0)	46.7 (45.3, 48.0)
Age		
18 to 24 years old	1,248 (17.3)	9.6 (9.0, 10.0)
25 to 44 years old	2,790 (38.6)	35.0 (33.5, 37.0)
45 or more years old	3,187 (44.1)	55.4 (53.7, 57.0)
Race/ethnicity		
Hispanic	910 (12.6)	10.0 (9.3, 11.0)
Non-Hispanic White	4,852 (67.2)	74.7 (73.4, 76.0)
Non-Hispanic Black	994 (13.8)	9.8 (9.0, 11.0)
Others	469 (6.5)	5.5 (4.8, 6.0)
Education		
Less than Bachelor’s degree	5,890 (81.5)	78.7 (77.4, 80.0)
Bachelor’s or advanced degree	1,335 (18.5)	21.3 (20.0, 23.0)
Employment status		
Full-time	3,227 (44.7)	46.5 (44.7, 48.0)
Part-time	1,270 (17.6)	15.3 (14.2, 16.0)
unemployed	2,728 (37.8)	38.2 (36.4, 40,0)
Household income		
Less than $50,000	4,988 (69.0)	61.1 (59.4, 63.0)
$50,000 or more	2,237 (31.0)	38.9 (37.3, 41.0)
Health insurance coverage		
Yes	5,758 (79.7)	83.3 (82.3, 84.0)
No	1,467 (20.3)	16.7 (15.7, 18.0)
Emergency room visit		
Yes	2,317 (32.1)	28.5 (26.9, 30.0)
No	4,908 (67.9)	71.5 (69.9, 73.0)

Data are sociodemographic characteristics of adult respondents (who were followed up across all six waves) at Wave 1 of the PATH study.

N, number of observations; CI, confidence interval.

### 3.2 CC/EC use patterns and behavior

Data regarding current CC and EC use status of respondents at baseline of each wave are presented in Table 2. Across waves, non-current use of CC/EC was the predominant behavior. The weighted proportion of non-current users was similar across waves, ranging from 52.8 to 56.7% of respondents. Initially, a downward change in non-current use was seen from Wave 1 to 2, but this began climbing after Wave 2. The rate of dual CC/EC use was similar over time as well, from 4.2% (weighted) of respondents at Wave 1 to 5.0% at Wave 5. Current exclusive EC use increased over time, from 2.2% of respondents at Wave 1 to 3.8% at Wave 5. In contrast, the proportion of exclusive CC smoking declined over time, starting from 39.4% at Wave 1 and going down in 34.5% at Wave 5.

**Table 2:** Current smoking and e-cigarette use status.

	Wave 1		Wave 2		Wave 3		Wave 4		Wave 5		
	*N* (%)	Weighted % (95% CI)	*N* (%)	Weighted % (95% CI)	*N* (%)	Weighted % (95% CI)	*N* (%)	Weighted % (95% CI)	*N* (%)	Weighted % (95% CI)	*p* value
Current use status											<0.001
Exclusive CC smoking	3,948 (54.6)	39.4 (38.2, 41.0)	3,883 (53.7)	39.1 (37.8, 40.0)	3,823 (52.9)	38.7 (37.1, 40.0)	3,791 (52.5)	38.4 (36.6, 40.0)	3,434 (47.5)	34.5 (33.1, 36.0)	
Dual use of CC and EC	420 (5.8)	4.2 (3.7, 5.0)	522 (7.2)	5.2 (4.6, 6.0)	452 (6.3)	4.5 (4.1, 5.0)	418 (5.8)	4.1 (3.6, 5.0)	509 (7.0)	5.0 (4.6, 6.0)	
Exclusive EC use	218 (3.0)	2.2 (1.9, 3.0)	261 (3.6)	2.9 (2.6, 3.0)	286 (4.0)	3.1 (2.7, 4.0)	260 (3.6)	2.9 (2.5, 3.0)	330 (4.6)	3.8 (3.3, 4.0)	
Non-current use	2,639 (36.5)	54.2 (52.9, 55.0)	2,559 (35.4)	52.8 (51.4, 54.0)	2,664 (36.9)	53.7 (52.1, 55.0)	2,640 (36.5)	54.7 (53.0, 56.0)	2,952 (40.9)	56.7 (55.2, 58.0)	

Data are unweighted counts, prevalence, and weighted prevalence of use behaviors at Waves 1–5 (baseline of the five 2-wave periods) among adult respondents who were followed up across all six waves.

CC, combustible cigarette; EC, e-cigarette; N, number of observations; CI, confidence interval. Exclusive CC smoking: current established CC smokers without using ECs currently at a given wave; Dual use of CC and EC: using CCs and ECs concurrently at a given wave; Exclusive EC use: current established EC users without smoking CCs currently at a given wave.

### 3.3 Associations between CC/EC use patterns and outcome variables over a 2-wave period

**Figure 1:**
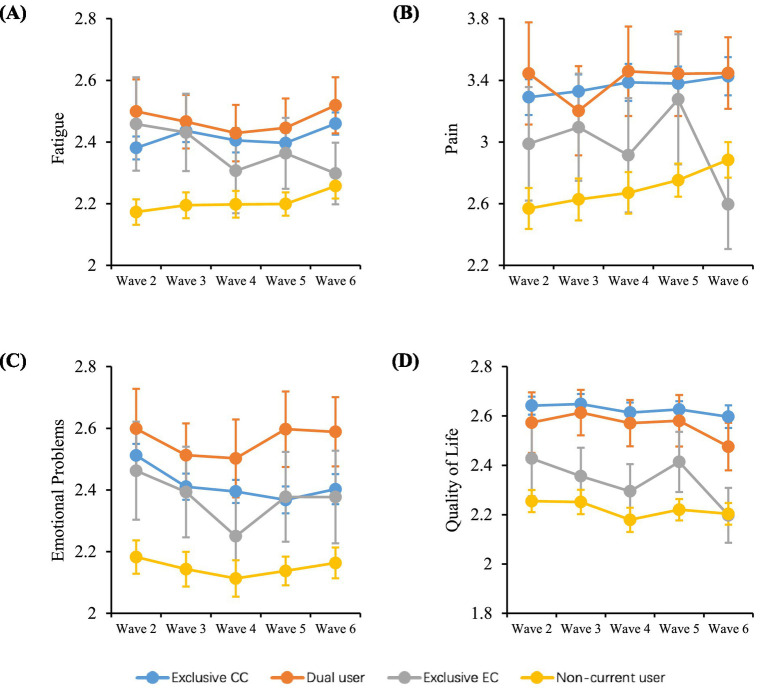
Changes in health-related symptoms and QoL over time. Data shown are unadjusted mean (±95% CI) scores over time for **(A)** fatigue, **(B)** pain, **(C)** emotional problems, and **(D)** QoL by different smoking and vaping behaviors among U.S. adults from Wave 2 (2014) to Wave 6 (2021). CC, combustible cigarette; EC, e-cigarette; QoL, quality of life. Exclusive CC smoking, current established CC smokers without using ECs currently at a given wave; Dual use of CC and EC, using CCs and ECs concurrently at a given wave; Exclusive EC use, current established EC users without smoking CCs currently at a given wave; Health-related symptoms include fatigue, pain and emotional problems: scores for pain range from 1 to 10, while scores for other symptoms range from 1 to 5, with a lower score indicating less symptom burden; QoL scores range from 1 to 5, with a lower score indicating better quality of life.

Assessments were made on the associations of dual use, exclusive EC use and non-current use of CC or EC behaviors compared with exclusive CC smoking with the outcome variables of fatigue, pain, emotional problems and QoL. Data from mixed-effects regression models, both unadjusted and adjusted for a number of covariates, are presented in Table 3. In addition, Figure 1 illustrates the average crude outcome variables for each pattern of CC and EC use over time. Generally, respondents who neither smoked CCs nor used ECs currently had the lowest mean scores for outcome variables, which was apparent in each two-wave follow-up. Exclusive EC users displayed the second lowest mean scores for outcome variables among the four groups. Health-related scores were comparable between dual users of CC and EC and exclusive CC smokers. Dual users exhibited the highest mean scores for fatigue, pain, emotional problems across waves, while exclusive CC smokers had the highest mean scores for QoL across waves (Table 2).

**Table 3:** Weighted longitudinal associations between current CC and EC use status and health-related symptoms and QoL over five 2-wave periods.

	Unadjusted		Adjusted	
	Mean difference (95% CI)	*p* value	Mean difference (95% CI)	*p* value
Fatigue				
Exclusive CC smoking	Ref.	Ref.	Ref.	Ref.
Dual use of CC and EC	0.019 (−0.023, 0.061)	0.379	0.012 (−0.029, 0.054)	0.561
Exclusive EC use	−0.071 (−0.128, −0.015)	0.013	−0.065 (−0.121, −0.009)	0.023
Non-current use of both	−0.112 (−0.138, −0.086)	<0.001	−0.089 (−0.115, −0.063)	<0.001
Pain				
Exclusive CC smoking	Ref.	Ref.	Ref.	Ref.
Dual use of CC and EC	0.041 (−0.068, 0.151)	0.460	0.094 (−0.016, 0.203)	0.093
Exclusive EC use	−0.278 (−0.428, −0.129)	<0.001	−0.206 (−0.355, −0.058)	0.006
Non-current use of both	−0.359 (−0.431, −0.287)	<0.001	−0.330 (−0.401, −0.258)	<0.001
Emotional problems				
Exclusive CC smoking	Ref.	Ref.	Ref.	Ref.
Dual use of CC and EC	0.029 (−0.019, 0.078)	0.236	0.015 (−0.033, 0.063)	0.542
Exclusive EC use	−0.023 (−0.088, 0.043)	0.497	−0.027 (−0.092, 0.038)	0.411
Non-current use of both	−0.095 (−0.125, −0.064)	<0.001	−0.070 (−0.101, −0.040)	<0.001
Quality of life				
Exclusive CC smoking	Ref.	Ref.	Ref.	Ref.
Dual use of CC and EC	−0.015 (−0.054, 0.024)	0.448	−0.001 (−0.039, 0.038)	0.968
Exclusive EC use	−0.125 (−0.178, −0.073)	<0.001	−0.103 (−0.155, −0.051)	<0.001
Non-current use of both	−0.182 (−0.207, −0.157)	<0.001	−0.165 (−0.190, −0.140)	<0.001

Data are unadjusted and adjusted mean differences (95% CI) in scores for fatigue, pain, emotional problems and QoL associated with dual use, exclusive EC use, and non-current use, compared with exclusive CC smoking.

CC, combustible cigarette; EC, e-cigarette; QoL, quality of life; CI, confidence intervals; ref, reference. Exclusive CC smoking, current established CC smokers without using ECs currently at a given wave; Dual use of CC and EC, using CCs and ECs concurrently at a given wave; Exclusive EC use, current established EC users without smoking CCs currently at a given wave; Fatigue and emotional problems were scored 1–5, while pain was scored 1–10: lower scores indicate less symptom burdens; QoL was scored 1–5: lower score indicates better quality of life; Wave pairs assessed were Wave 1-Wave 2, Wave 2-Wave 3, Wave 3-Wave 4, Wave 4-Wave 5 and Wave 5-Wave 6; Adjusted mean differences were obtained from linear mixed-effects regression (LMER) models after controlling for age, gender, race/ethnicity, level of education, employment status, household income, health insurance, emergency room visit, individual-level effect and wave.

With the adjusted regression model and compared with exclusive CC smokers, dual use of CCs and ECs did not exhibit a significant association with any of the four outcome variables, i.e., the mean differences were not statistically significant, as shown in Table 3. In contrast, exclusive EC use was found to significantly reduce fatigue and pain, as well as improve QoL (Table 3). On average, respondents who exclusively used ECs experienced a decrease of approximately 0.065 units in mean fatigue score (95% CI: [-0.121, -0.009]), 0.206 units in mean pain score (95% CI: [-0.355, -0.058]), and 0.103 units in mean QoL score (95% CI: [-0.155, -0.051]), respectively, compared to exclusive CC smokers. It is also noted that exclusive EC use did not have a significant effect on emotional problems scores when compared to exclusive CC smoking (adjusted mean difference: −0.027, 95% CI: [−0.092, 0.038]). Furthermore, non-current use of CCs or ECs resulted in significant reductions in pain, fatigue, emotional problems, as well as improvement in QoL, after a two-wave period, in both the adjusted and unadjusted models (Table 3). Specifically, non-current use of CCs or ECs was associated with an estimated decrease of 0.089 units in average fatigue score (95% CI: [-0.115, -0.063]), 0.33 units in average pain score (95% CI: [-0.401, -0.258]), 0.07 units in average emotional problems score (95% CI: [-0.101, -0.040]), and 0.165 units in average QoL score (95% CI: [-0.190, -0.140]).

## 4 Discussion

In this study, using data from the nationally-representative PATH study we confirm that quitting smoking is associated with both improved general QoL and lower levels of health symptoms related to QoL such as pain, fatigue, and emotional problems, which are consistent with the results of previous studies (43–46). In addition, we add to the previous findings and demonstrate that exclusive EC use, but not dual use, was associated with improved general QoL, as well as reduced fatigue and pain, but not with reduced emotional problems. Interestingly, our findings align with previous data showing that complete switching, but not necessarily dual use, may reduce toxicant exposure (14–20), is associated with beneficial changes in some biomarkers of potential harm (25–28, 42), and thus may improve smokers’ general health.

These findings are important when taking into account the proportion of smokers who are able to quit unaided compared with those who are able to quit using ECs. Quitting smoking unaided is inherently difficult, and increasing evidence supports that the likelihood of making a successful quit attempt is significantly higher when ECs are used to support smoking cessation compared with unaided quitting (30, 32–34). In addition, during the early stages of quit attempts, reductions in QoL may promote relapse and lead to failed cessation attempts (43). Overall, our findings give rise to the possibility that ECs may support smoking cessation and prevent relapse by increasing QoL and reducing factors which can influence QoL such as fatigue and pain. Additionally, our findings also suggest that QoL and related factors should be taken into account when attempting to determine the full harm reduction potential of ECs.

In another analysis of PATH study data, focusing on those respondents who were cancer survivors (i.e., who self-reported a history of cancer), fatigue, pain, emotional problems and general QoL were higher among current smokers than among former and never smokers. Fatigue, pain, and emotional problems, were also higher among current EC users compared with non-users (47), although that particular finding contrasts with other findings suggesting that both multi-factorial assessments of QoL (48), as well as individual assessments of general QoL (48), were not different between EC users and non-users. In the study by Price et al. (47), general QoL and associated factors were not examined between ex-smoking current EC users and current smokers. Furthermore, the studies by Price et al. (47) and Ridner et al. (48) used cross-sectional approaches and did not assess changes in QoL and associated factors over time. In another study, improvements in 6MWD, a factor which may be indicative of, and correlates with, QoL in patients with chronic lung disease (55), were observed among COPD patients in a longitudinal study who switched to using ECs for 24 and 36 months (51, 52). Overall, our findings concur with these previous longitudinal study findings, while adding novel insight by being one of the first studies to examine changes in QoL among the general population with various patterns of CC and EC use.

Our study has several strengths. First, we utilized nationally-representative survey datasets comprising a substantial numbers of respondents to comprehensively assess changes in health status indicators and self-perceived QoL associated with different patterns of CC and EC use. Second, the longitudinal design of the study, facilitated by the repeated outcome measures available through the PATH Study, allowed us to examine the potential long-term effect of EC use patterns on subsequent health-related problems and QoL over a prolonged period of approximately 8 years.

The findings of our analyses of PATH data, however, are subject to some limitations. First, participants who have quitted or switched for a longer period of time may have different symptom burdens or QoL scores compared to those who recently quitted or switched, as well as those who have just initiated CC or EC use. Unfortunately, due to the absence of CC/EC use information between successive waves, our study was unable to precisely determine the smoking/vaping cessation or particular switching status, nor could it consider these behaviors and adjust these variables accordingly. Additionally, the reliance on self-reported health-rated outcomes from the PATH data could introduce the possibility of recall bias. Second, our study examined, similar to prior research (47), 4 variables related to health-related outcomes and QoL of different smoking or vaping statuses. However, we did not assess patients with specific diseases such as cancer (47), and further studies would be necessary to ascertain whether CC/EC use patterns have the potential to improve fatigue, pain, emotional problems and QoL in individuals suffering from smoking-related disease. Third, health-related symptom burdens and QoL may influence an individual’s decision-making regarding tobacco product use. For example, those reporting diminished QoL may receive advice from healthcare professionals to reduce CC consumption or to transition to EC use. Meanwhile, individuals experiencing significant symptoms, such as pain, may seek to change their product use behavior to alleviate their discomfort. This potential for reverse causality makes it difficult for us to draw definitive conclusions about causal effect, despite our use of a longitudinal assessment (56). Another limitation is the use of a single, general question on QoL from the PATH Study as one of the primary outcomes. Future studies could assess the impact of CC/EC use patterns on more specific health status domains associated with QoL by administering more detailed instruments such as the 36-item short-form health survey (SF-36) (57) which along with many other domains also assesses those examined in this study, the US Centers for Disease Control and Prevention Health-Related Quality of Life (HRQOL) instrument (58, 59), or the tobacco use-specific Tobacco Quality of Life Impact Tool (TQOLIT) (45) and the Smoking Cessation Quality of Life questionnaire (SCQoL) (43, 44). Moreover, assessment of health status and QoL using instruments specific to known smoking related diseases such as COPD (60) may also be of interest, especially since smokers with COPD who switched to using ECs experienced improvements in 6MWD (51, 52), a potential contributing factor to QoL in those suffering from COPD. To this point, single-item measures may be lacking in reliability and validity when compared with disease-specific instruments (61).

## 5 Conclusion

In summary, smokers who neither smoked CCs nor used ECs or used ECs exclusively had less health-related symptoms in some health-related domains and better QoL than those who were exclusive CC smokers. While the harm reduction potential of ECs is generally assessed by reductions in the risk of developing smoking-related diseases such as heart disease and cancer, this impact on QoL should also be taken into account when assessing their harm reduction potential. Furthermore, the development of practices to help smokers switch and improve their QoL also needs to be considered.

## Data Availability

Publicly available datasets were analyzed in this study. This data can be found here: Population Assessment of Tobacco and Health (PATH) Study [United States] Public-Use Files (ICPSR 36498): https://www.icpsr.umich.edu/web/NAHDAP/studies/36498/datadocumentation.
